# Supplementary material for: Splice-Junction-Based Mapping of Alternative Isoforms in the Human Proteome
Source: Cell Rep. Author manuscript; Available in PMC 2020 Jan 15. (PMC6961840; doi:10.1016/j.celrep.2019.11.026)
Supplement: 2 [file NIHMS1546469-supplement-2.zip › DF1/PXD010154/Spleen/Spleen_17_PEX1_VTLISQAMFPCGILSTSAGGGGAAVTVAFTNAR.pdf]

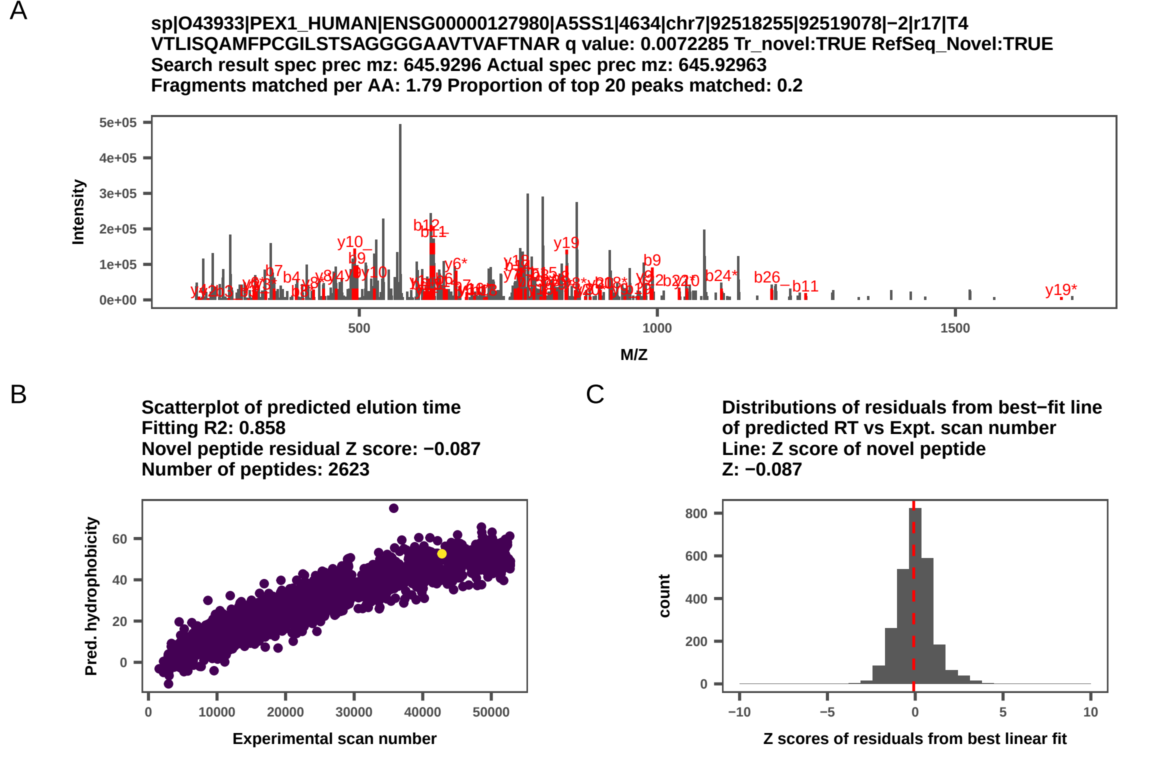
A
sp|O43933|PEX1_HUMAN|ENSG00000127980|A5SS1|4634|chr7|92518255|92519078|−2|r17|T4
VTLISQAMFPCGILSTSAGGGGAAVTVAFTNAR q value: 0.0072285 Tr_novel:TRUE RefSeq_Novel:TRUE
Search result spec prec mz: 645.9296 Actual spec prec mz: 645.92963
Fragments matched per AA: 1.79 Proportion of top 20 peaks matched: 0.2
Intensity
5e+05
4e+05
3e+05
2e+05
1e+05
0e+00
M/Z
500
1000
1500
b12
y10
y19
y6*
b9
b24*
b26
b11
y19*
B
Scatterplot of predicted elution time
Fitting R2: 0.858
Novel peptide residual Z score: −0.087
Number of peptides: 2623
Pred. hydrophobicity
60
40
20
0
Experimental scan number
0
10000
20000
30000
40000
50000
C
Distributions of residuals from best−fit line
of predicted RT vs Expt. scan number
Line: Z score of novel peptide
Z: −0.087
count
800
600
400
200
0
Z scores of residuals from best linear fit
−10
−5
0
5
10
